# Artificial intelligence in orthopedic surgery: evolution, current state and future directions

**DOI:** 10.1186/s42836-022-00112-z

**Published:** 2022-03-02

**Authors:** Andrew P. Kurmis, Jamie R. Ianunzio

**Affiliations:** 1 Discipline of Medical Specialties, University of Adelaide, Adelaide, SA Australia; 2 Department of Orthopaedic Surgery, Lyell McEwin Hospital, Vale, Elizabeth, SA Australia; 3 School of Medicine, University of Adelaide, Adelaide, SA Australia

**Keywords:** Artificial intelligence, Arthroplasty, AI, Machine learning

## Abstract

Technological advances continue to evolve at a breath-taking pace. Computer-navigation, robot-assistance and three-dimensional digital planning have become commonplace in many parts of the world. With near exponential advances in computer processing capacity, and the advent, progressive understanding and refinement of software algorithms, medicine and orthopaedic surgery have begun to delve into artificial intelligence (AI) systems. While for some, such applications still seem in the realm of science fiction, these technologies are already in selective clinical use and are likely to soon see wider uptake. The purpose of this structured review was to provide an understandable summary to non-academic orthopaedic surgeons, exploring key definitions and basic development principles of AI technology as it currently stands. To ensure content validity and representativeness, a structured, systematic review was performed following the accepted PRISMA principles. The paper concludes with a forward-look into heralded and potential applications of AI technology in orthopedic surgery.

While not intended to be a detailed technical description of the complex processing that underpins AI applications, this work will take a small step forward in demystifying some of the commonly-held misconceptions regarding AI and its potential benefits to patients and surgeons. With evidence-supported broader awareness, we aim to foster an open-mindedness among clinicians toward such technologies in the future.

## Introduction

The incorporation of technology into everyday medical practice is accelerating at an incredible rate — in few areas more so than in the domain of orthopedic surgery. Real-time navigated, computer-guided [1] and robot-assisted [1, 2] intraoperative input has become commonplace in many regions. Two-dimensional imaging is rapidly being replaced by virtual three-dimensional (3D) displays [3–5], and interactive digital, semi-automated or fully-automated preoperative planning and templating are also widely available in many developed settings [3].

While the logic-driven computing processes that underpin such technologies are impressive to say the least, most output functions are the result of ‘human-defined’ iterative pathways with parameters set in keeping with progressive logic principles. The integration of artificial intelligence (AI) machine learning (ML) algorithms into driving decision-making pathways represents an evolution beyond the basic limitations of conscious human learning considerations and accepted logistic analytical regression [6, 7].

Once a realm of science fiction, AI applications have rapidly integrated into accepted ‘everyday’ life around us. Many are surprised to learn for how long ‘everyday’ examples of AI intrusion into our worlds has been commonplace. The AI-driven ‘Google Search’ function came into mainstream use nearly 25 years ago (1998) [8], predicting search patterns and ‘pre-empting’ active searching. In a more contemporary sense, ‘Google Translate’ [9], Facebook’s ‘Phototagger’ (2015) [8], Uber’s rideshare demand prediction, and Apple’s well-known voice-responsive pocket assistant ‘Siri’ [9] are all examples of mature AI algorithms with wide public application.

While orthopedic surgeons have long stood proudly behind the craftmanship of their clinical trade, we can no longer remain ignorant to the global forward creep of technology. As we seek to further improve the quality and outcomes of the services we provide, computer-assisted and AI applications show increasingly apparent opportunities for their integration into everyday practice [1]. The exponentially-expanding volume of information we collect pre-, intra- and post-surgery, and the coupled unprecedented data aggregation rate [10, 11], lends itself almost perfectly to offloading to computer-driven applications [1, 10, 12, 13]. While patient-generated health data (PGHD) [14] are becoming commonplace, the sheer enormity of captured data points for a single patient [15–17] yield almost more information than can perceivably be comprehended and managed by the human processing capacity. Even ‘off-the-shelf’ wearable sensors [15, 16] may capture several million discrete data points [14] for small cohorts, or single patients tracked for extended time periods. This mass of stored digital information is collectively known as ‘big data’ [8, 10, 12, 18, 19]. Computers (and computer applications) are ideally suited to objectively receive, categorize and interpret such large amounts of patient- and care-related materials. However, before orthopedic surgeons can confidently relinquish responsibility of data management to computers and overcome the innate biases towards such a process, one must have at least a basic understanding of the potentially advantageous role of computers in life, healthcare and surgery, including how such technologies have evolved, what they are reasonably suited to do, and how we quality-assure their contributions moving forward. The aim of this review is to offer, to the practising clinical orthopedic surgeons, definitions and fundamental understanding of AI and its applications, with the goal of demystifying some of the elements and misconceptions that harbour an oppressive reluctance to engage with technology in our working spheres. It is certainly not the intent to provide an all-encompassing and highly-detailed summary, rather to ‘introduce’ the key topic elements to the naïve reader.

## Methods

**Fig. 1:**
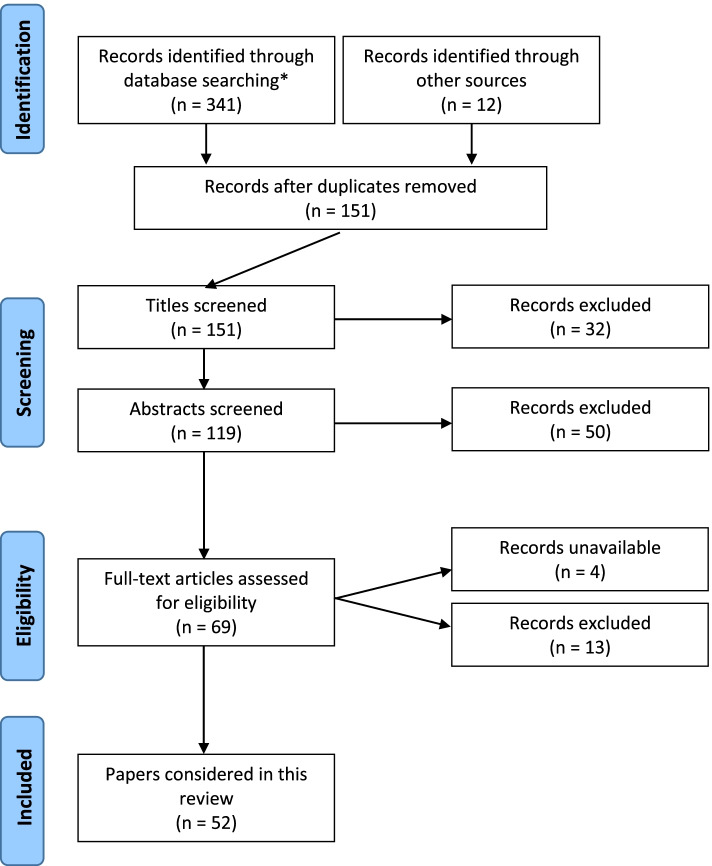
PRISMA search summary. * Tues Aug 31 03:05:102021 Search: ((artificial intelligence OR AI) AND (orthopaedics OR orthopedics) AND (arthroplasty OR joint replacement) AND (TKR OR THR OR THA OR TKA) AND (English\[Language]))

To ensure a relevant, accurate and representative synopsis of the current state of understanding of AI in orthopedics, a formal, structured and systematic search and retrieval of publications was performed according to the accepted Preferred Reporting Items for Systematic Reviews and Meta-Analyses (PRISMA) guidelines. The search results are depicted in Fig. 1. Three databases: (i) Cochrane, (ii) EMBASE and (iii) Medline, were searched from inception until 31 August 2021. Search results were limited, in the first instance, to articles available in the English language with available abstracts.

Initially, 353 articles were identified during preliminary database searching. After exclusion of duplicates, articles which did not match the search intent (*i*.*e*. papers not specifically exploring AI applications) and articles not available in full text form, 65 full-text papers were manually reviewed. At the end of the review process, 52 articles were deemed appropriate for inclusion. As a relatively new topic in the field of medicine, it was found that there existed a lack of quantitative research within the domain AI in surgery, thus preventing formal ‘meta-analysis’ *per se*. With the preserved intent of providing a contemporary synopsis of the topic, a structured review of the identified literature was performed in keeping with meta-synthesis principles.

### Artificial intelligence

The term ‘artificial intelligence’ was coined by John McCarthy in 1956 [8, 11], originally as a theoretical proposition of a *future* stage whereby computers would ‘learn’ to perform automated tasks through algorithmic pattern recognition with limited (if any) direct human input [8]. In a more practical sense, AI has represented a state of ‘cognitive unloading’ whereby a computer can be tasked with the activity of scrutinizing large volumes of data in time frames uncomprehendable by the conscious human mind. These computers follow programmed algorithms (sets of computational instructions) to perform highly specific tasks. This may include ‘categorizing’, ‘identifying’ or ‘linking’ discrete variables, and specific factors may be selectively weighted more or less favorably [13] in decision-making analysis. The practical value of such task delegation becomes the ability to sift through large volumes of information in rapid time [20–22]. Depending on the assigned ‘task’, the computer(s) may identify trends or ‘patterns’ [8] in the data set which can be used to demonstrate attribute association. In its simplest form, the computer is offered a ‘training’ dataset [3, 23] (often a subset of a larger volume of known information). In clinical orthopedic practice, training datasets will routinely contain information pertaining to several thousand individual patients [21, 24]. With highly-specified coding (instructions), the computer ‘learns’ [25] to recognize specific features. The accuracy with which the computer can do this is often then compared to a known ‘gold standard’ (historically a human-defined standard). Large volumes of data can be fed into the AI system which ultimately seeks to ‘pattern recognize’ with great speed and reproducibility, usually linking to a pre-determined outcome or series of outcomes. The accuracy of the system can be progressively refined as the algorithm is adjusted to facilitate progressively more accurate assessment of the dataset. The new outcome is again measured against a gold standard, the accuracy determined, and error improvement measures feed back into the index algorithm. In this sense, the AI system is ‘taught’ to be more precise/accurate. This process has been likened to the process of progressive human learning whereby sequential exposure and re-visiting reinforces understanding.

The AI training cycle can be repeated (usually many times) with sequential algorithm amendments until an acceptable level of precision is achieved. Each full learning cycle (referred to as an ‘epoch’) allows progressive refinement of the discriminatory AI algorithm, improving the system accuracy. Depending on the size and complexity of the training dataset, and the desired final system ‘accuracy’ requirements, many conventional AI systems will be looped through between 10 and 1000 epochs [7, 23, 26, 27]. This process of ‘teaching’ the computer to perform a semi-automated task is referred to as ‘machine learning’ [10, 12, 13, 28–30]. While for certain tasks a highly accurate and reproducible outcome can be achieved, this process is usually time- and human-input-intensive to achieve initial establishment. Once a ‘final’ algorithm has been settled upon (with precision deemed acceptable to the task at hand), the AI system can be applied independently to future (previously unseen) datasets and, through predictive modelling [31], be used to determine a likely outcome based on complex multivariate analysis.

As a practical example, AI algorithms have been applied to arthroplasty component recognition from plain radiographs [32]. The algorithm is given information regarding key imaging features that a ‘human’ interpreter might use to discern one type or brand of implant from another. This might include information regarding length/size, proximal body *versus* stem segment proportions, changes in curvature, neck-shaft angles, the presence or absence of a collar *etc*. A training set of images is presented to the system (*i*.*e*. a series of plain radiographs of implants for which the actual brand/model is known). The AI system then assesses the images trying to match radiographic features to known (inputted) implant parameters. After this, the process of image assessment [26] has been completed, the ‘accuracy’ of the system is established *versus* the known (correct) answers. Where the system has been inaccurate, additional detail/information can be fed back into the algorithm (i.e. manually entered) and the training epoch repeated. This cyclic process can be repeated – with sequential addition of information to the algorithm – until a level of accuracy deemed ‘acceptable’ is achieved. The AI system is then ready for ‘real life’ application.

**Fig. 2:**
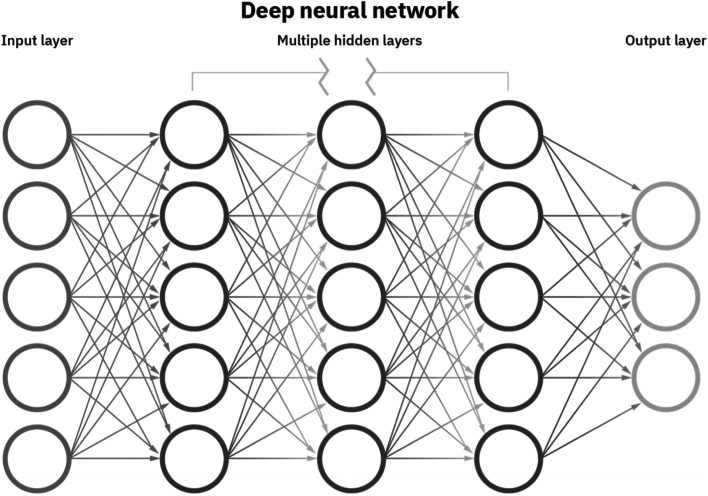
A stylized deep neural network depiction. Source: https://www.ibm.com/cloud/blog/ai-vs-machine-learning-vs-deep-learning-vs-neural-networks

With progressive computational power and algorithm refinement capacity, the AI system can be ‘taught’ to self-evaluate its own performance and amend its own internal algorithmic codes to improve performance [8]. The index AI algorithm refines with growing exposure to training datasets, sequentially improving iteration accuracy and ultimately maximizing predictive power [13]. While this seems simply a more refined version of the previously described ML processes, it requires an entirely different iterative programming basis whereby the program has capability and autonomy to ‘self-write’ its own coding instructions, a step towards true ‘automation’ [33, 34]. In doing so, it eliminates the need for direct human input/involvement in the algorithm refinement process and can greatly reduce the timeframe required to achieve a viable/usable system. This version of AI is referred to as ‘deep learning (DL)’ [10, 20, 23, 25, 32]. The system starts with a set of pre-determined key outcomes and known, linked, associative variables. It progressively re-refines its cluster association capacity with each new epoch, improving accuracy. The algorithmic functionality of modern DL neural networks [3] allows the artificial establishment of multi-layered ‘evolutionary plexuses’ that have been conceptually likened to the human neurons [11]. Most DL systems consist of some form of artificial neural network (ANN) [22, 25, 28], a series of iterative processing steps between an ‘input’ layer (for example where the data being considered are entered) and a final ‘output’ layer. A stylized depiction is shown in Fig. 2. Each initial input variable or data point can be linked to one or more (or all) evaluative steps in the next layer. In turn, each discrete data point in the second layer can be linked to one or more (or all) evaluative steps in the next layer, and so on. These layers between the initial input and final output layers are referred to as ‘hidden layers’. The larger the number of input points, and the larger the number of neural ‘hidden layers’, the greater the complexity of analysis (and the greater the demand upon computer processing power). With increasing sophistication of inter-relation, data points embedded within individual layers can be influenced in a selectively disproportionate manner by adjacent data points (in a sense, the influence of these points can be weighted more heavily in influencing the final output) — such complex systems are commonly referred to as deep convolutional neural networks [CNNs] [26, 35–37].

There are many potential advantages to such true DL ‘artificial intelligence’ (beyond the value of timeliness), including being free from the otherwise incumbent limitations of human inconsistency, fatigue [8], cognitive judgement error [8], selection bias and poor inter-user correlation — all of which plague manual (human) assessment of large-volume multi-focal datasets. Dealing with such enormous sets of information, of almost infinite complexity [9], AI systems have the potential to identify variable associations that have not previously been considered [15] in an evidence-based ‘manual’ manner — in a sense, amplifying human cognition [9]. Many argue that this is where the ‘true value’ of AI in healthcare may lie in the future (inter-related risk factor association to desired or known clinical endpoints is an obvious exemplar). As appealing as this may sound, current AI systems are equally not without their limitations and inherent problems. Beyond the need for huge processing capacity (and associated expense), AI systems are currently only as good as the human processes that establish them. The ‘garbage in, garbage out’ mantra holds true here. If the index AI algorithms are set up with intrinsic errors or fundamental mis-assumptions, these will be propagated through the process and result in erroneous outputs. The further the system self-refines, the greater the sequential error can become. While the system’s primary objective may be set to achieve outcome ‘accuracy’, it may internally achieve this by refinement changes that violate basic human logic principles to best ‘achieve’ its desired task.

### Essential requirements for AI

In essence, there are three basic prerequisites for the development of an AI system. Firstly, a large volume of data pertinent to the topic of interest must exist and be able to be inputted in some predictable and consistent manner to the system itself. Data entry and/or extraction are often underappreciated key elements of the AI process. Imprecise or unrepresentative data provision will ultimately lead to erroneous determinants and outputs. Secondly, substantial computing and data processing power (capacity) is needed [8, 12, 19]. In many iterations, this has been the rate-limiting step to the forward advancement of AI applications. As discussed previously, as the number of data input sources and the number of ‘hidden layers’ increase – especially within a deep CNN – the demand on computer processing capacity increases almost exponentially. Computer ‘processing’ capacity is commonly measured in units known as ‘Floating Operations Per Second (FLOPS)’. In comparing the most powerful computer processors from 1955 to 2015, there has been a one-*trillion*-fold increase in FLOPS [19]. As a more practical example, the computer processing unit (CPU) of the entire Apollo 11 moon landing guidance computer in 1969 was estimated to be approximately 2 MHz; by comparison, a pocket-held iPhone 12 personal mobile phone has a CPU speed on 3.1 GHz (*i*.*e*. 3100 MHz) – that being a more than 1500 times greater processing power.

The third requirement of an AI system is an underlying algorithm. Given the wide diversity of AI applications (well beyond the closed sphere of medicine), different AI algorithms have evolved to best manage specific problems [38]. While a detailed synopsis falls beyond the scope of this paper, a basic summary of common AI algorithms is shown in Table 1. While many (if not all) of these algorithmic subtypes have conceivable application in medicine and orthopedic surgery specifically, ‘Association Rule Learning Algorithms’, ‘Deep Learning Algorithms’ and ‘Artificial Neural Network Algorithms’ are perhaps the most commonly encountered ones to date.

**Table 1:** Common AI algorithm types

Algorithm type	Definition
**Regression Algorithms**	Centred around basic statistical principles and adopted early within ML frameworks. Usually used to predict one variable based on the known value of other variables. Used often in areas that require numerical estimation such as forecasting and trend analysis.
**Decision Tree Algorithms**	Represents a decision pathway based on comparison to a known set of pre-determined ‘rules’. Application of each rule occurs at a ‘node’. Depending on the determined answer, the decision is progressed to the next linked node, and another answer is determined. The nodes therefore ‘branch’ with sequential layers of assessment from a starting point – hence the name ‘decision tree’. For example, ‘does the feature of interest have A or B characteristics? If B, does the feature then have C or D characteristics? If C, does the feature then have E characteristics?’, and so on. Complexity increases as the number of interconnected decision-making steps increases.
**Clustering Algorithms**	Is an approach based around grouping features of interest into relatively homogenous classes. This is done based on recognized element similarities. Are often used for preliminary data analysis and the isolation of like subpopulations. These smaller cohort fragments can then be separately explored for identifiable within-group commonalities.
**Instance-Based Algorithms**	Does not require ‘training’, *per se*, rather stores a series of exemplars in memory and compares new instances to these with the goal of establishing a ‘best match’ based on similarity. Each new case is analysed independently and can often be time consuming. Often work best in instances whereby the target function is complex but can be simplified into less complex generalisations.
**Association Rule Learning Algorithms**	Is a common means for initial data mining of relatively ‘raw’ datasets. It involves analysis of specific attributes looking for repetitive dependencies (*i*.*e*. What precursor features or elements are consistently associated with an observed outcome?) Often used to determine cause and effect relationships between critical events captured within the dataset. When linked with Bayesian theorem, event or outcome probability prediction can often be achieved with high reliability.
**Ensemble Algorithms**	Is an umbrella term to describe the practice of using multiple independently trained ‘weaker learning algorithms’ and merging the combined analysis output. Highly susceptible to inaccuracies within the individual algorithms combined (much akin to the integrity of a systematic literature review being undermined by included poor quality RCTs). Performed well however, is regarded as one of the most effective and ‘powerful’ algorithmic styles.
**Artificial Neural Network (ANN) Algorithms**	As discussed previously, ANNs are interconnected iterative sequences based conceptually upon human (biological) neural networks. They are commonly used for regression and classification. Acknowledged to be an extremely complex analytical subfield, consisting of many variations and algorithms for specific problems. Usually time-consuming to establish and require high computer processing capacity. A rapidly growing field both within and outside medicine.
**Deep Learning (DL) Algorithms**	Is currently the newest form of neural networks employed in healthcare. Use large modelling domains with a complex and hierarchical structure usually composed of many interconnected, nonlinear ‘layers’. Have been applied with great effect in areas such as image and feature recognition (see ‘face recognition’ technologies in the lay world and ‘diagnosis’ from digital imaging within medicine). Deep convolutional neural networks represent an evolved DL platform centred around the established mathematical principle of convolution which considers the fluid impact of one variable interacting with another to generate a third, separate function.

### Potential advantages of AI in orthopedics

The are many potential advantages to the incorporation of AI into everyday orthopedic practice. There are conceivably diagnostic, decision-making and technique execution, and administrative considerations. Prior to surgery, AI applications have already been employed to either improve the accuracy of, or reduce the time associated with critical diagnostic steps. This may involve recognition and classification of pathology, or the correct determination of *in situ* implant types/models. The excellent discriminative capacity [9] of digital image-based AI applications lends itself to such uses. This has clear and immediate implications for revision surgery whereby accurate identification of the current patient implant (often reflecting models no longer in routine use) is critical for salvage option planning and equipment ordering. Several high-quality papers have already been published in this domain reporting AI system precision in the preoperative image-based identification on *in situ* hip and knee replacement constructs. Such papers described ‘near-perfect’ functionality [20, 27] with accuracies as high as 99.6% [23] or better [35] claimed in the correct identification of limited series of included implant types. Additionally, all of the available clinical applications in automated implant recognition cited significant time-saving during this process [20–22]. Murphy* et al*. (2021) reported an average implant recognition time of just 0.96 s using an out-dated off-the-shelf iPhone 6 after photographing the relevant patient anteroposterior (AP) pelvic radiographs [22]. This incredible time-saving is already being touted as an enormous potential cost-saver through reducing the time otherwise spent by clinicians to perform such similar ‘manual identification’ tasks [20]. There are many more such examples of ‘real life’ clinical applications of AI technologies which are explored in the next section.

Common decision-making activities can also be supported by, or offloaded to, AI applications. In the setting of a well-established and informed AI system, the ability to associate and consider a myriad of linked patient variables provides, in many circumstances, superior risk prediction [39], which can be used prospectively to achieve evidence-supported risk mitigation [6]. Similarly, by assessment or incorporation of postoperative patient-reported outcome measure (PROM) data, AI algorithms can be trained to pattern-recognize key elements (singularly or multi-factorially) which contribute to optimized clinician- and/or patient-defined outcomes. Used appropriately, this information can be looped back into patient screening and decision-making processes and may be helpful in preoperative expectation management [28] and allowing provision of objective feedback regarding the quality of delivered care [33]. Both considerations may be used to ultimately improve patient outcomes [24, 29, 35]. Inextricably linked to this prospective risk-determination capacity is the ability to subgroup patients based on perceived risk profile, allowing episode-of-care cost prediction [18, 25, 28]. Such analyses have already been applied to arthroplasty cohorts of group sizes approaching 150,000 patients [undergoing total knee arthroplasty (TKA)] [18].

From an information management perspective, AI algorithms have already been shown to hold great value. The two key elements of improved data management accuracy and time saving (largely through amelioration of the need for a human time commitment) are central here. Both have undeniable potential for considerable healthcare cost savings [20]. As patient medical records transition more and more uniformly to electronic based medical records (EMRs), the burden of near-oppressive data gathering volumes plagues many developed health networks. With so much volumetric data available, how does a single clinician sort through and retrieve key elements critical for point-of-care decision making? Published forays into EMR data synthesis and extraction have shown very positive potential [21]. Such applications – after only modest epoch training cycles — have been shown to provide higher key information extraction accuracy than ‘manual’ record searching, but with far greater speed [33]. A recently published paper by van de Meulebroucke and colleagues (2019) showed an impressive accuracy of over a 95% in EMR data extraction using AI in a cohort of nearing 550 patients in a non-academic healthcare setting [33].

### Clinical applications

Despite being still considered by many a ‘novel’ field, AI in orthopedic surgery is being used with ever-increasing frequency. Large volumes of patient data, ever-increasing patient expectations of positive post-surgical outcomes, and a profession-driven push to improve the quality and precision of care we offer have opened many avenues for AI use. Orthopedic applications include image recognition (diagnostics/implant identification [32]), risk prediction, cost-outcome determinations, clinical decision making seem popular early targets of AI technologies. Applications in primary TKA [7], primary total hip arthroplasty (THA) and resurfacing [23], and primary total shoulder arthroplasty (TSA) [18, 28] have all been reported with positive value.

In a preoperative sense, AI has been used and reported in areas such as: length-of-stay [18, 25, 28] and episode-of-care cost prediction, each with reportedly ‘excellent’ validity [18]. Grading of osteoarthritis from plain radiographs was shown in early versions to be as accurate as fellowship-trained arthroplasty surgeons in making the same determination, but significantly quicker [37]. As to pathologic feature recognition [8, 27] (including fractures), its precision has been further improved by recursive feature elimination [40] whereby training datasets can be refined to allow more ‘targeted’ feature-of-interest recognition (thus reducing image feature ‘noise’ that may otherwise degrade the accuracy of human interpretation), 3D templating and operative planning [3] having achieved accuracy of 90% or greater compared to just 56.7% for conventional acetate-based manual planning and the patient-specific influence of pelvic sagittal inclination [34] on THA construct stability being able to be determined to inform intraoperative acetabular component placement. Discharge destination prediction [25, 28, 41], the likelihood of prolonged opioid prescription after THA [6], and the likelihood of blood transfusion after TKA [42] have also been successfully reported. Even more user-friendly web-based applications [41, 42] for discharge destination and RBC transfusion likelihood have also been tested in live clinical scenarios.

The automated image processing [27] capacity of AI has also been applied to the diagnosis of periprosthetic joint infection [24] against the Musculoskeletal Infection Society (MSIS) standard; peri-prosthetic fracture classification [21] *as per* the Vancouver classification, with purported sensitivity of 100 and specificity of 99.8%; and diagnosis of periprosthetic component loosening [36] of both THA and TKA constructs, with an overall accuracy of 88.3%. Postoperatively, the overall prospective determination of the likelihood of future need for TKA revision has also been demonstrated with high utility in a large cohort of 25,104 patients after the primary procedure [7], as ha the value in automated postoperative monitoring and outcome assessment [13], risk prediction [8], including the likelihood of dislocation after primary THA [26]. Ultimately, applications already in research or early clinical use have been shown to predict [40, 43, 44] and/or improve patient satisfaction [45] and overall outcomes [19].

### Looking into the future

The principal value of AI appears to hinge around data-driven optimiszed outcomes [12]. The technology has already been shown to have value in supporting [9, 29, 39, 45] or driving clinical decision making [8, 13]. The feedback loop incorporation of PROMs [14, 29] continues to help improve both subjective patient satisfaction and clinical outcomes [19, 45]. Large studies have already been completed after hip and knee replacement surgery exploring the minimum clinically-important differences in multiple standard PROMs [13, 46] to better inform the differentiation between ‘statistical’ and ‘actual’ clinical differences. With increasing refinement of complex discriminative AI algorithms, one could anticipate this to improve in the future. Pre-emptive risk profiling for potential perioperative adverse events or major complications [39] after arthroplasty surgery will also help to inform patient-centric consenting and material risk determination. Such robust preoperative information will undoubtedly be utilized to drive more patient-individualized reimbursement and episode-of-care payment models [8].

As medicine and surgery push onward towards a seemingly inevitable uptake of technology-assisted data management (including potentially AI applications), there presents a unique opportunity for a more universal commitment to ‘standardization’ of prospective patient data capture and storage. This will facilitate future seamless integration of EMRs with PGHD systems [14] — and subsequent data extraction — to allow optimized *post-hoc* data management and analysis. The current inconsistencies and incompatibilities in fundamental computing platform and operating systems stand as a key barrier to potentially more meaningful uptake. While ‘open access’ platforms would facilitate greater data usability, in their current form, such systems lack the intrinsic patient privacy and confidentiality requirements necessary to support wider healthcare use. The opportunity for further work in this realm clearly exists such that protection of patients’ interests and rights keep pace with the desire for technological advancement.

## Discussion

Artificial intelligence applications are already in widespread use in medicine and the sub-fields of orthopedics and have already shown great potential to transform care [8, 10]. The ability of refined algorithms to draw upon digital information readily stored in large database and registry [7, 18, 25, 28, 30] repositories further improves the value, accuracy and practical relevance of the outcomes reported. Particularly self-determining systems, such as the newer deep CNNs [26, 35–37], have increasing capacity for the management of complex datasets [9] and may allow the identification of variable associations not previously conceived through conventional human-driven analytics. Certainly others involved in the development of such technologies are enthusiastic about the future and those immersed in such current clinical applications espouse AI as holding very real potential to expand the horizons of orthopedics [12, 26].

Much still needs to be done, however, before AI applications can be generally accepted into mainstream care [11]. In the current setting, the computing hardware requirements alone carry great intrinsic expense and within global healthcare systems under ever-increasing cost pressure the burden of utilitarianistic parsimony [29] remains an often rate-limiting road block. Many novel ‘research’ applications still require verification and validation in the wider clinical setting [3] or at least result duplication remote from the index institution(s). Many impartial observers concede only a role for AI applications in augmenting clinical decision-making, not yet being in a position to replace it [36] — in many areas, this likely holds true.

It is important to note that, with some applications, preliminary research has suggested no value over conventional approaches. For example, the recent 2020 publication by Pau and colleagues [30] suggested that AI self-learning algorithms did not outperform simple logistic regression in predicting postoperative walking limitation after joint replacement surgery. Similarly, a large registry-based AI analysis in Denmark failed to show a meaningful benefit in the application of ML algorithms to the prediction of future early revision need following primary TKA [7], despite using four different, purpose-designed AI models. The authorship group did concede, however, that their iterative logistic regression approaches may have lacked the necessary embedded comorbidity data required to allow sufficient predictive value and demonstrate a ‘true’ benefit.

## Conclusions

As technology advances further in the world around us, the role that AI applications play in orthopedics is already seen by many as an inevitable future step. Much investment and early work have already explored the optimization of early utilization of such approaches, across a range of clinical and para-clinical domains. Like any new technology, effort must be expended to ensure AI applications touted for clinical use have shown evidence-based rationales for adoption with non-inferiority (but ideally improved) outcomes against the conventional standards. It is important that the science underpinning such advancements is not outpaced by the hype. While it is likely that computer- and AI-based programs will add value to areas where human cognition and capacity stand as rate-limiting factors, the expense and effort required to establish such systems must be positively weighed against the perceivable benefit. Current generation AI algorithms, particularly deep convoluted CNNs, lend themselves to image feature recognition and multi-variate risk analysis/outcome prediction and these are the current areas of greatest research interest. Volumetric data management and prospective episode-of-care/payment model stratification are also being actively explored. Being such a novel and unprecedented frontier, AI in medicine presently lacks widely-accepted governance and regulatory provisions which will need to evolve at a similar rate to ensure safe and optimal utilization with respect for individual patient data and circumstances.

## Data Availability

Not applicable.

## Abbreviations

3D: Three dimensional
AI: Artificial intelligence
ANN: Artificial neural network
AP: Anteroposterior
CNN: Convolutional neural network
CPU: Computer processing unit
DL: Deep learning
EMR: Electronic medical record
FLOPS: Floating Operations Per Second
ML: Machine learning
MSIS: Musculoskeletal Infection Society
PGHD: Patient generated health data
PRISMA: Preferred reporting items for systematic reviews and meta-analyses
PROMs: Patient-reported outcomes measures
TKA: Total knee arthroplasty
THA: Total hip arthroplasty
TSA: Total shoulder arthroplasty
